# Supplementary material for: Heatwave-Related Mortality Risk and the Risk-Based Definition of Heat Wave in South Korea: A Nationwide Time-Series Study for 2011–2017
Source: Int J Environ Res Public Health. 2020 Aug 7;17(16):5720. doi: 10.3390/ijerph17165720 (PMC7460278; doi:10.3390/ijerph17165720)
Supplement: Supplementary file 1 [file ijerph-17-05720-s001.pdf]

Supplementary Materials

Title: Heatwave-related mortality risk and the risk-based definition of heat wave in South Korea: A nationwide time-series study for 2011–2017

Author names and affiliations

Cinoo Kang[1], Chaerin Park[1], Whanhee Lee[1], Nazife Pehlivan[1], Munjeong Choi[1], Jeongju Jang[1], Ho Kim[1,2*].

Cinoo Kang and Chaerin Park contributed equally to this research as co-first authors.

[1]Department of Public Health Science, Graduate School of Public Health, Seoul National University, Seoul, Korea.

[2]Institute of Health and Environment, Seoul National University, Seoul, Korea

Contents

Supplementary Tables (Table S1 to S5)

Supplementary Figure (Figure S1)

Supplementary Tables

Table S1. Details information of data collection for urbanization indicators.

Indicator	Collection Period	Distribution organization	Survey organization
Population	2011-2016	Database of community health outcomes and health determinants	Ministry of Government Administration and Home affairs
Percentage of aged 65 y+ population	2011-2016		Ministry of Government Administration and Home affairs
Number of beds in hospitals per 1,000 persons	2011-2015		Ministry of Interior and Safety
Number of physicians per 100,000 persons	2011-2016		National Health Insurance Service
Number of hospitals	2016		National Health Insurance Service
Percentage of total urban forest area	2011, 2013, 2015		Korean Forest Service
Local income tax	2008[1]	Korean Statistical Information Service	Ministry of Interior and Safety

[1] Because there were three areas merged or newly created to municipal areas, only three districts had different collection period; Cheongju-shi: 2014, Changwon-shi: 2010, Sejong-shi: 2012.

Table S2. Descriptive statistics of urbanization relevant indicators and climatic variables in the total, urban, and rural areas; values: mean (min, max) value (summer during 2011-2017).

	**Total (229 districts)**	**Urban (147 districts)**	**Rural (82 districts)**
Population[1]	2.2 (0.1, 11.5)	3.2 (0.4, 11.5)	0.5 (0.1, 2.1)
Number of beds in hospitals (per 1,000 persons)	13.1 (0.1, 53.3)	13.6 (0.1, 48.3)	12.4 (0.1, 53.3)
Proportion of total urban forest area (%)	41.5 (1.8, 420.3)	36.9 (3.0, 420.3)	49.7 (1.8, 188.3)
Local income tax[2]	48092.1 (640.0, 859538.2)	59802.7 (1315.8, 859538.2)	27098.7 (640.0, 280818.9)
Climatic variables			
Mean temperature (ºC)	23.3 (19.1, 24.9)	23.7 (19.1, 24.9)	22.7 (20.0, 24.3)
Range of daily mean temperature (ºC)	17.6 (14.2, 27.4)	17.8 (14.8, 27.4)	17.3 (14.2, 21.5)

[1]Unit: 100,000.
[2]Currency: won; unit: 1,000,000.

Table S3. Sensitivity analysis results on lag effect days and the degrees of freedom(df) for seasonality and lag under 2 different definitions of heat wave duration.

**Conditions**			**Duration[1] ≥ 2**			**Duration[1] ≥ 3**		
Seasonality[2]	Lag[3]	Lag knots[4]	Threshold (%)	Max (%)	RR (95% CI)	Threshold (%)	Max (%)	RR (95% CI)
2	10	2	93	99	1.13 (1.04, 1.23)	93	99	1.11 (1.03, 1.18)
3	10	2	93	99	1.10 (1.01, 1.20)	93	97	1.08 (1.02, 1.14)
4	7	2	94	98	1.12 (1.07, 1.18)	91	98	1.08 (1.03, 1.14)
4	14	2	94	99	1.14 (1.02, 1.27)	93	98	1.09 (1.02, 1.17)
4	10	3	93	98	1.10 (1.03, 1.16)	93	98	1.08 (1.02, 1.14)

[1]Heatwave duration (consecutive days exposed to heatwave).
[2]Degrees of freedom for day of season (flexibility of seasonality and overall temporal trends).
[3]Lag effect days (lagged responses between heatwave and mortality considering shorter and longer lag day effects).
[4]Degrees of freedom for lag (flexibility of lag response curve).

Table S4. Descriptive statistics of total deaths and other urbanization relevant indicators in the total, urban, and rural areas; values: mean (min, max) value (summer during 2011-2017).

	**Total (229 districts)**	**Urban (147 districts)**	**Rural (82 districts)**
Total deaths[1]	590639 (148, 10929)	493911 (344, 10929)	96728 (148, 2561)
Percentage of aged 65 y+ population	17.0 (5.8, 34.8)	12.6 (5.8, 25.9)	24.9 (10.5, 34.8)
Number of physicians (per 100,000 persons)	2.4 (0.8, 21.9)	2.8 (0.8, 21.9)	1.8 (0.9, 5.5)
Number of hospitals	344.3 (11, 2898)	434.8 (31, 2898)	179.8 (11, 805)

[1]Values of total deaths are sum (min, max).

Table S5. Percentile of the distribution of mean temperature for the daily maximum temperatures at 33 ºC and 35 ºC in the total, urban, and rural areas during the summer season (June to September) of the study period (2011-2017).

	Mean (Standard deviation)		
	**Total (229 districts)**	**Urban (147 districts)**	**Rural (82 districts)**
**33 ºC (32.5-33.5 ºC)**	96.18 (3.26)	96.10 (3.30)	96.35 (3.18)
**35 ºC (34.5-35.5 ºC)**	98.44 (2.02)	98.45 (1.81)	98.43 (2.42)

Supplementary Figure

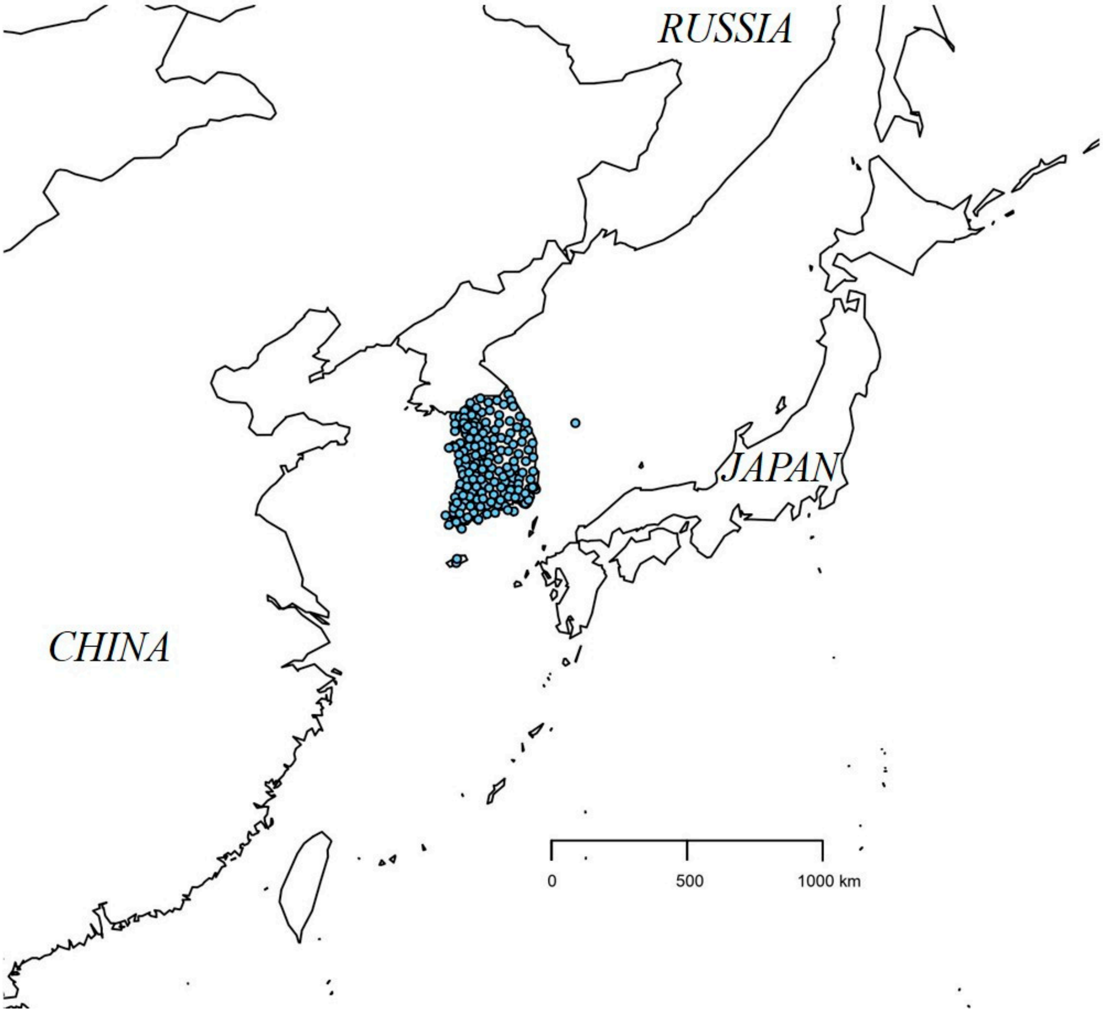

Figure S1. Geographical location of South Korea
